# Can increased vigilance for chronic kidney disease in hospitalised patients decrease late referral and improve dialysis-free survival?

**DOI:** 10.1186/s12882-018-0869-6

**Published:** 2018-04-02

**Authors:** M. De Wilde, M. Speeckaert, W. Van Biesen

**Affiliations:** Department of Nephrology, Ghent University Hospital, De Pintelaan 185, 9000 Ghent, Belgium

**Keywords:** Care path, Chronic kidney disease, Dialysis-free survival, Early referral, Follow-up, Mortality, Vigilance

## Abstract

**Background:**

Insufficient vigilance for renal insufficiency is associated with late referral, increased morbidity and mortality. The present study examines whether increased vigilance for chronic kidney disease (CKD) leads to quicker referral to and better follow-up by a nephrologist, and whether it is associated with an improved outcome.

**Methods:**

Patients with an eGFR < 45 ml/min/1.73 m^2^ during hospitalisation at the Ghent University Hospital were enrolled during a period of 100 days. The patients were interviewed about their awareness of CKD. Both the patients and their general practitioner were subsequently informed about CKD. The primary endpoint was the number of patients referred for nephrological follow-up within three months. The secondary endpoint was need for dialysis and mortality from any cause one year after inclusion.

**Results:**

Of the 72 included patients, 54 had proven CKD, with eGFR consistently < 45 ml/min/1.73 m^2^ during at least three months before inclusion. Merely 65% was aware of having CKD and only 41% was in regular nephrological follow-up. After intervention, the percentage of patients with CKD in follow-up increased from 41% to 71% (*p* = 0.002). The proportion reaching the secondary endpoint was significant lower in the patients who were referred quickly than in those who were not (*p* = 0.015). Similarly, the proportion was significant lower in the patients who received nephrological follow-up than in those who did not (*p* = 0.006).

**Conclusion:**

Vigilance for CKD is poor. Simple interventions to augment the vigilance for CKD, as presented in this study, lead to a quicker referral to and follow-up by a nephrologist, which may result in better outcome.

## Background

Early detection, follow-up and treatment of CKD patients reduce the morbidity and mortality and delay the progression of renal failure [1, 2]. Two thirds of the patients with CKD stages 3, 4 or 5 require one or more medical interventions or therapy adjustments, including a better control of arterial hypertension, correction of anaemia, management of mineral bone disorders or discontinuation of nephrotoxic medications [3–7].

Active participation of patients in their own care is critical [8, 9], but may be limited by the lack of awareness and understanding of CKD [10], ensuing in late referral [9]. A late referral to a multidisciplinary nephrology team is associated with a worse outcome after the start of dialysis and decreases the access to kidney transplantation [11]. Longer and more intense nephrological follow-up of patients who eventually undergo dialysis is commensurate with better survival [9, 11–13]. In Belgium, a care path for chronic renal insufficiency was developed in 2009 by the National Institute for Health and Disability Insurance, in order to tackle CKD in a multidisciplinary way and increase the vigilance for CKD. Nevertheless, screening for renal insufficiency is presently still substandard, even in patients at risk [11]. The vigilance for renal insufficiency is still insufficient in general practitioners [14–16] as well as in medical specialists [5, 17], leading to delayed diagnosis and late referral to a nephrologist [18], which can result in inadequate treatment [16, 19, 20] with possible impact on morbidity and mortality [21].

The goal of the present study was to examine if an increased vigilance for CKD in hospitalised patients results in a quicker referral to the nephrology outpatient clinic, a better follow-up and finally an improved outcome. More specifically, we explored whether there was a difference in dialysis-free survival and mortality rate.

## Methods

The present study was a single center study performed in the Ghent University Hospital (Belgium). Subjects were enrolled over a period of 100 days, between November 2013 and February 2014. Patients, hospitalised on a selected number of medical wards in the hospital (thoracic and vascular surgery, cardiac surgery, cardiology, endocrinology, infectious diseases, dermatology and geriatrics) with an eGFR < 45 ml/min/1.73 m^2^ (according to CKD-EPI), were identified on a daily basis by a computer query program. The following exclusion criteria were used: patients with advanced dementia, patients in a palliative situation or having an end-stage organ failure, patients receiving any form of renal replacement therapy (peritoneal dialysis, haemodialysis or kidney transplantation) and patients with acute kidney injury (AKI), documented by a recent blood analysis, taken less than 3 months before identification by the computer program, showing an eGFR > 60 ml/min/1.73 m^2^.

All patients provided informed consent. An eGFR measurement predating the current test for at least 3 months was obtained through search in the health records or by contacting the general practitioner to define the CKD status of the patient. Patients were interviewed about their awareness of CKD, follow-up by a nephrologist and inclusion in a care path. Patients and their general practitioner were subsequently informed about renal insufficiency, the presence of CKD and the possibility of inclusion in a care path. Three and twelve months after inclusion in the study, we checked if the patient had received a consultation on the nephrology outpatient clinic and whether a care path was initiated by contacting their general practitioner and/or nephrology outpatient clinic. In addition, the evolution of the kidney function and the outcome (initiation of dialysis and mortality from any cause) were recorded.

The primary endpoint of this study was whether the increased vigilance for CKD led to a higher number of patients referred to a nephrology outpatient clinic (within three months), receiving follow-up by a nephrologist and included in a care path. The secondary endpoint of the study was a combined endpoint of need for dialysis and mortality from any cause, reached between three months and one year after inclusion in the study.

Statistical analyses were performed using MedCalc (MedCalc, Ostend, Belgium). Differences between groups were evaluated using the Student’s t-test and the Fisher’s exact test with a two-tailed *p*-value. A *p*-value < 0.05 was considered a priori to be statistically significant.

## Results

**Fig. 1:**
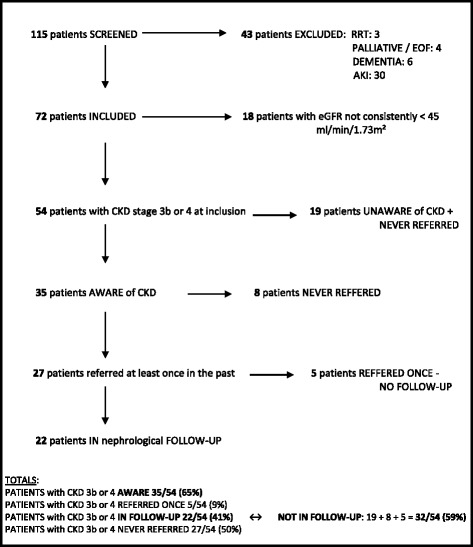
Patient inclusion and vigilance for CKD. RRT, number of patients who received renal replacement therapy at time of inclusion; PALLIATIVE / EOF, number of patients in a palliative stage or with end organ failure at time of inclusion; DEMENTIA, number of patients with dementia at time of inclusion; AKI, number of patients with acute kidney injury

During the inclusion period, 115 patients were identified with an eGFR < 45 ml/min/1.73 m^2^*(*Fig. 1*)*. A total of 43 patients were excluded: 3 patients already received dialysis, ten patients had advanced dementia, an end-stage organ failure or received palliative care and 30 patients had AKI. The final cohort thus consisted of 72 patients (51 males and 21 females, mean age: 76.6 ± 8.5 years) with a mean eGFR of 33.9 ± 6.9 ml/min/1.73 m^2^.

Fifty-four patients (54/72) had proven CKD with an eGFR consistently < 45 ml/min/1.73 m^2^ during at least three months before inclusion, of which 36 had CKD stage 3b (eGFR: 30–45 ml/min/1.73 m^2^) and 18 CKD stage 4 (eGFR: 15–30 ml/min/1.73 m^2^). Only 65% (35/54) of patients with CKD stage 3b or 4 were aware of suffering from renal failure. Half of the patients with CKD stage 3b or 4 (27/54) were never referred to a nephrologist, 9% (5/54) was once referred to a nephrologist before but was no longer in follow-up, and only 41% (22/54) was followed by a nephrologist on a regularly basis. The number of patients who consulted a nephrologist at least once (13/18 vs 14/36; *p* = 0.042) or who were still followed by a nephrologist (11/18 vs 11/36; *p* = 0.042) was significantly higher in the group of patients with CKD stage 4 in comparison with CKD stage 3b, respectively. In the group of 32 patients with CKD, who were not followed by a nephrologist before inclusion, only 22% (7/32) was visited by a nephrologist during their hospitalization on request of their treating physician. In five patients (5/54) follow-up after inclusion was not possible due to completion of the secondary endpoint or a palliative stage within three months after inclusion. None of them was in follow-up by a nephrologist before inclusion.

Eighteen patients (18/72) did not had a proven advanced CKD at the time of inclusion, but were possibly evolving into a CKD stage 3b. 16/18 patients had never consulted a nephrologist before and had no knowledge of any renal impairment. In six patients (6/18), follow-up after inclusion was not possible due to completion of the secondary endpoint or a palliative stage within three months after inclusion. Of the remaining 12 patients, six evolved to a CKD stage 3b.

**Fig. 2:**
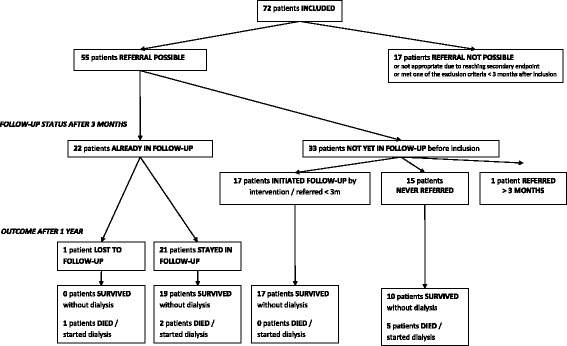
Referrral status after 3 months and outcome after 1 year

Three months after inclusion *(*Fig. 2*)*, 55 patients (mean age: 76.7 ± 8.0 years, 69% males) were diagnosed with CKD (mean eGFR: 34.5 ± 7.0 ml/min/1.73 m^2^). Seventeen patients (17/72) could not be referred or receive follow-up due to completion of the secondary endpoint or meeting one of the exclusion criteria within three months after inclusion. Thirty three (33/55) patients were not yet followed by a nephrologist before their inclusion. The kidney function of the CKD patients who were not yet followed by a nephrologist before inclusion was significantly higher than the eGFR of the 22 patients who were already in follow-up (36.9 ± 5.8 ml/ min/1.73 m^2^ vs 30.9 ± 7.1 ml/min/1.73 m^2^, *p* = 0.001). More than half of these patients (17/33), consulted a nephrologist within three months after inclusion and started a care path. The proportion of patients in follow-up increased significantly from 41% (22/54) to 71% (39/55) within three months after inclusion (*p* = 0.002), an increase with 74%. One patient consulted a nephrologist and started a care path six months after inclusion. The other fifteen patients (15/33) did not visit a nephrologist.

Comparison of the outcome one year after inclusion in CKD patients, not yet followed by a nephrologist before, shows that the need to start dialysis and/or mortality rate was significantly lower in the CKD patients who were referred quickly, stayed in follow-up and started a care path, compared with CKD patients who did not (0/17 vs 5/15; *p* = 0.015). The compared groups did not differ significantly in gender, age, eGFR, number of patients with a therapy restriction or number of patients who stayed in a home care facility *(*Table 1*)*. In addition, the need to start dialysis and/or mortality rate one year after inclusion was significantly lower in CKD patients who were followed at the nephrology department compared with the CKD patients who did not (2/38 vs 6/16; *p* = 0.006). Again, the compared groups did not differ significantly in gender, age, eGFR, number of patients with a therapy restriction or number of patients who stayed in a home care facility *(*Table 2*)*.

**Table 1:** Characteristics and outcome of patients who were referred < 3 months after inclusion vs patients who were never referred

	Referred < 3 months + follow-up	Not referred - No follow-up	*P*-value
Total number of patients	17	15	
eGFR (mean)	38.6 (+/− 4.4)	35.8 (+/− 6.0)	*p* = 0.151
Age (mean)	74.9 (+/− 8.4)	78.7 (+/− 8.0)	*p* = 0.224
Males/females	12/5	10/5	*p* = 1.00
Lives in care facility: Yes/No	0/17	3/12	*p* = 0.092
Therapy restriction: Yes/No	2/15	2/13	*p* = 1.00
Need of dialysis or died within 1 year after inclusion: Yes/No	0/17	5/10	*p* = 0.015

**Table 2:** Characteristics and outcome of patients who were in follow-up with a nephrologist vs patients who were not

	Follow-up	No follow-up	*P*-value
Total number of patients	38	16	
eGFR (mean)	34.6 (+/− 6.9)	34.8 (+/− 7.2)	*p* = 0.931
Age (mean)	75.6 (+/− 8.0)	78.9 (+/− 8.1)	*p* = 0.168
Males/females	27/11	10/6	*p* = 0.540
Lives in care facility: Yes/No	1/37	3/13	*p* = 0.073
Therapy restriction: Yes/No	4/34	2/14	*p* = 1.00
Need of dialysis or died within 1 year after inclusion: Yes/No	2/36	6/10	*p* = 0.006

## Discussion

This study reveals that in patients admitted to a tertiary care hospital, CKD is unacknowledged in a substantial number of patients. These data are supported by previous findings in European populations. In the UK, nearly 85% of patients with CKD, defined as a serum creatinine of > 2.03 mg/dl in men and > 1.53 mg/dl in women, were not in follow-up [4]. In Northern Ireland, only a minority of patients with a serum creatinine > 1.7 mg/dl were referred to a nephrologist [22]. Also in Germany, relatively few patients (39%) with CKD-stage 4 were followed with a nephrologist [23].

Vigilance for renal insufficiency increases significantly in more advanced CKD stages. Among patients with CKD stage 3b, 69% was not followed by a nephrologist vs 39% among patients with CKD stage 4. The observed difference in vigilance for CKD according to the CKD stage is consistent with the literature [19]. More than a third of the CKD patients in the present study were not aware of their renal insufficiency, although the kidney function was measured at previous time points. This is in line with the results of a British study, where 31% of the patients with CKD stage 3B, 4 or 5 were unaware of their disease [3].

The underlying reasons why advanced CKD remains out of scope in so many patients are presumably multifactorial (1): several guidelines concerning when to refer to a nephrologist offer conflicting information [24] or are subject to national [25, 26] or regional [27] related measures and agreements (2), these guidelines are often insufficiently known by clinical practioners [17, 19, 21, 28, 29] and (3) the evidence of the published guidelines is rather scarce.

This study has succeeded to detect patients with advanced CKD not yet followed by a nephrologist, and to inform this group of patients and their general practitioners about renal insufficiency, the presence of CKD and the possibility for inclusion in a care path. In this way, the vigilance for renal insufficiency was augmented, creating a higher alertness for conditions interacting with a declining kidney function. In slightly more than half of the newly discovered patients with advanced CKD, a care path was initiated after discharge, leading to a significant augmentation of the number of patients with CKD in follow-up by a nephrologist.

In patients where the intervention did not lead to follow-up by a nephrologist, a significant higher need for dialysis and/or increased mortality rate in the year after inclusion was observed. This is in line with previous literature. In a prospective British study, CKD patients (serum creatinine ≥2.03 mg/dl in men and ≥ 1.53 mg/dl in women; equivalent to an eGFR < 43 ml/min/1.73m^2^ and a mean eGFR of 28.5 ml/min/1.73m^2^) who were not followed by a nephrologist, showed a significantly higher mortality [4]. In a retrospective study of CKD patients, defined as a serum creatinine of > 1.4 mg/dl, a better survival of patients with CKD stage 3 and 4 was noted in those who received nephrology follow-up [30]. A similar finding was found in diabetic veterans with CKD stage 3 and 4 [31].

The majority of general practitioners feel that they have not received adequate training regarding time or indications for referral of patients with progressive kidney failure [32]. Increasing the vigilance for renal insufficiency as presented in this study is trying to provide a higher level of involvement and information, in both the patient and their physicians, with the goal of promoting compliance and follow-up.

Despite the fact that there was no statistically difference in age, gender or eGFR between the compared groups, despite the exclusion of patients with advanced dementia and patients in a final palliative situation or with an end-stage organ failure as well as the exclusion of patients who achieved the secondary endpoint during the first three months after inclusion, a selection bias could still be responsible for the difference in achieving the secondary endpoint. Nevertheless, the number of patients with a therapy restriction and the number of patients who stayed in a home care facility, did not differ significantly between these groups. The major limitation of the study is its observational nature. Although demographic, clinical and biochemical characteristics were similar between the groups, it is conceivable that more compliant patients are more likely to present for further follow-up. Poor compliance is responsible for 42% of the late referrals [33] and may have contributed to the observed differences in survival. In addition, the decision by physicians not to refer the patient for nephrological follow-up is often practiced for reasons of existing co-morbidities [34]. Since we did not correct for all comorbid conditions, comorbidity may have further biased the results at the disadvantage of the patients that were not referred. A further limitation is the small sample size and the single center design of the study. The present findings should therefore be confirmed in a larger multicenter study.

## Conclusion

The vigilance for renal insufficiency of both general practitioners and medical specialists remains insufficient. By detecting and informing patients with advanced CKD, a higher awareness of the disease in both patients and physicians is obtained, leading to a significant quicker referral to and follow up by a nephrologist. The combined endpoint of mortality from any cause and the need for dialysis was significantly lower in patients who were referred early to the nephrology outpatient clinic and followed by a nephrologist.

## Abbreviations

AKI: Acute kidney injury
CKD: Chronic kidney disease
eGFR: Estimated glomerular filtration rate
